# Intranasal Insulin Prevents Anesthesia-Induced Spatial Learning and Memory Deficit in Mice

**DOI:** 10.1038/srep21186

**Published:** 2016-02-16

**Authors:** Yongli Zhang, Chun-ling Dai, Yanxing Chen, Khalid Iqbal, Fei Liu, Cheng-Xin Gong

**Affiliations:** 1 Jiangsu Key Laboratory of Neuroregeneration, Co-Innovation Center of Neuroregeneration, Nantong University, Nantong, Jiangsu 226001, China; 2 Department of Neurochemistry, Inge Grundke-Iqbal Research Floor, New York State Institute for Basic Research in Developmental Disabilities, Staten Island, New York 10314, United States of America; 3 Department of Neurology, the Second Affiliated Hospital, School of Medicine, Zhejiang University, Hangzhou 310009, China

## Abstract

Elderly individuals are at increased risk of cognitive decline after anesthesia. General anesthesia is believed to be a risk factor for Alzheimer’s disease (AD). At present, there is no treatment that can prevent anesthesia-induced postoperative cognitive dysfunction. Here, we treated mice with daily intranasal administration of insulin (1.75 U/day) for one week before anesthesia induced by intraperitoneal injection of propofol and maintained by inhalation of sevoflurane for 1 hr. We found that the insulin treatment prevented anesthesia-induced deficit in spatial learning and memory, as measured by Morris water maze task during 1–5 days after exposure to anesthesia. The insulin treatment also attenuated anesthesia-induced hyperphosphorylation of tau and promoted the expression of synaptic proteins and insulin signaling in the brain. These findings show a therapeutic potential of intranasal administration of insulin before surgery to reduce the risk of anesthesia-induced cognitive decline and AD.

It is known that elderly individuals are at increased risk of cognitive decline after general anesthesia^1^,^2^,^3^. Anesthesia may also be one of the contributing factors for sporadic Alzheimer disease (AD), a devastating neurodegenerative disease characterized clinically by cognitive deficit and also the most common form of dementia in adults. Some epidemiological studies have shown that general anesthesia may increase the risk of elderly individuals for developing AD^4^,^5^. Increased atrophy of the brain, including the cortical gray matter and the hippocampus, together with reduced performance for cognitive tests are detectable 5 to 9 months after general anesthesia/surgery^6^. Evidence from animal studies suggests that anesthetic exposure can increase Aβ plaque formation and tau hyperphosphorylation^7^,^8^,^9^,^10^,^11^, which are believed to cause neurodegeneration in AD. Anesthesia also causes learning and memory deficits at a later time in rodents^9^,^12^,^13^. Rats after exposure to isoflurane, a commonly used inhaled anesthetic, show learning impairment that persists for weeks^14^. Persistent memory impairment is also seen in aged rodents after exposure to isoflurane, nitrous oxide, or the combination of both^15^,^16^. The molecular mechanisms by which general anesthesia causes long-term cognitive impairment remain to be understood.

The major known function of insulin is to regulate glucose metabolism in the periphery. The brain was previously thought to be insensitive to insulin. However, recent studies have well demonstrated that insulin actually has neurotrophic and neuroprotective activities, regulates neural development and plasticity, and plays an important role in learning and memory^17^,^18^,^19^,^20^. The important role of brain insulin signaling is further supported by clinical studies showing that administration of insulin into the central nervous system promotes memory and improves cognitive function in individuals with AD^21^,^22^,^23^.

We recently found that general anesthesia disturbs brain insulin signaling and induces abnormal hyperphosphorylation of tau^24^, which might contribute to the anesthesia-induced cognitive impairment. We therefore hypothesized that administration of insulin into the brain may prevent anesthesia-induced brain changes and cognitive impairment. To test this hypothesis, we treated 3xTg-AD mice, a commonly used triple transgenic mouse model of AD, with insulin via intranasal delivery for consecutive seven days before anesthesia with propofol. Intranasal delivery bypasses the blood-brain barrier and delivers drugs into the brain through several pathways, including olfactory- and trigeminal-associated extracellular pathways and the perivascular pathway^25^. We found that the pretreatment of 3xTg-AD mice with intranasal insulin promotes brain insulin signaling and attenuates propofol-induced hyperphosphorylation of tau^24^. However, whether the pretreatment with intranasal insulin can also prevent anesthesia-induced cognitive impairment had not been studied. The present study aimed to answer this important question.

In our present study, we treated wild-type mice with daily intranasal administration of insulin or, as a control, saline for seven consecutive days before general anesthesia using a combination of propofol and sevoflurane. We found that intranasal insulin prevented anesthesia-induced spatial memory deficits. We also found that hyperphosphorylation of tau induced by anesthesia was temporary, which can be prevented with the insulin pretreatment.

## Results

### Intranasal insulin prevents anesthesia-induced spatial memory deficit in mice

**Figure 1:**
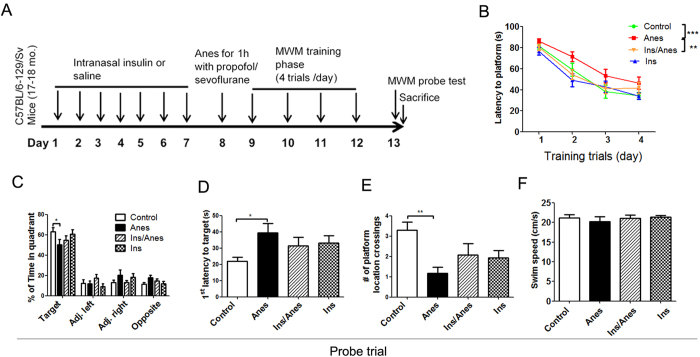
Effect of intranasal insulin treatment on anesthesia-induced deficit in spatial learning and memory in mice. **(A)** Animal study design. **(B)** Latency to locate the platform during training trials for 4 consecutive days (4 trials/day) in Morris water maze. (**C–F**) Probe trials performed 24 hrs after the last training trial. The percent time of mice in the target quadrant (**C**), the first latency to reach the platform location **(D)**, the number of the platform location crossing **(E)**, and the swim speed (**F**) of mice during the 60 sec. probe test are shown. Data are presented as mean ± SEM (n = 11–13 per group). These results indicate that anesthesia induces spatial learning and memory impairment and that intranasal insulin treatment can prevent this impairment.

We recently found that intranasal administration of insulin (1.75 U/day) for seven consecutive days promotes brain insulin signaling and attenuates propofol-induced hyperphosphorylation of tau in adult 3xTg-AD mice^24^. To investigate if the intranasal insulin treatment can also prevent anesthesia-induced cognitive impairment, we treated wild-type mice with intranasal insulin daily for seven days before general anesthesia, followed by assessment of spatial learning and memory using Morris water maze beginning at the next day after anesthesia (Fig. 1A). Anesthesia was induced by intraperitoneal injection of propofol (150 mg/kg) and maintained by 2.5% sevoflurane inhalation for 1.0 hr. The combination of these two anesthetics is commonly used in clinic for surgeries. We observed that all mice were able to learn the platform location during the training phase, as evidenced by the reduction in latency to locate the submerged platform from around 80 seconds in day 1 to 35–50 seconds in day 4 of the training (Fig. 1B). However, the anesthesia-treated mice (red curve) took significantly more time (*p* < 0.05) to locate the platform than the control mice (green curve), suggesting impaired learning in the anesthesia-treated mice. Importantly, we found that prior daily treatment with intranasal insulin for seven consecutive days prevented the anesthesia-induced learning impairment. The learning curve of the insulin-treated anesthesia group (orange curve) was significantly different from that of the untreated anesthesia group (red curve, *p* < 0.05) and was indistinguishable from the control mice (green curve, *p* > 0.05) (Fig. 1B). The intranasal insulin treatment did not affect the spatial learning of mice not treated with anesthesia (blue curve vs. green curve, *p* > 0.05).

To assess the spatial memory of the mice, we carried out probe trial 24 hrs after the last training trial (Fig. 1A). We found that the anesthesia group spent significant less time (50 ± 6% as compared to 63 ± 5% in controls, *p* < 0.05) in the target quadrant (Fig. 1C), took more time (39.5 ± 5.6 seconds as compared to 22.4 ± 2.7 seconds in controls, *p* < 0.05) to reach the platform location target (Fig. 1D), and crossed the platform location much less times (1.2 ± 0.3 times as compared to 3.3 ±0.5 times in controls, *p* < 0.05) (Fig. 1E) than the control mice. These results confirmed the spatial memory deficit in the anesthesia-treated mice. We found that prior daily treatment with intranasal insulin prevented the anesthesia-induced spatial memory deficit because no significant differences in these three parameters were found between the anesthesia- and insulin-treated group and the control group (Fig. 1C–E). These findings suggest that intranasal insulin treatment can prevent anesthesia-induced spatial learning and memory in mice. We also determined the swim speed and found no differences among the four mouse groups (Fig. 1F).

### Intranasal insulin attenuates anesthesia-induced hyperphosphorylation of tau in the mouse brain

**Figure 2:**
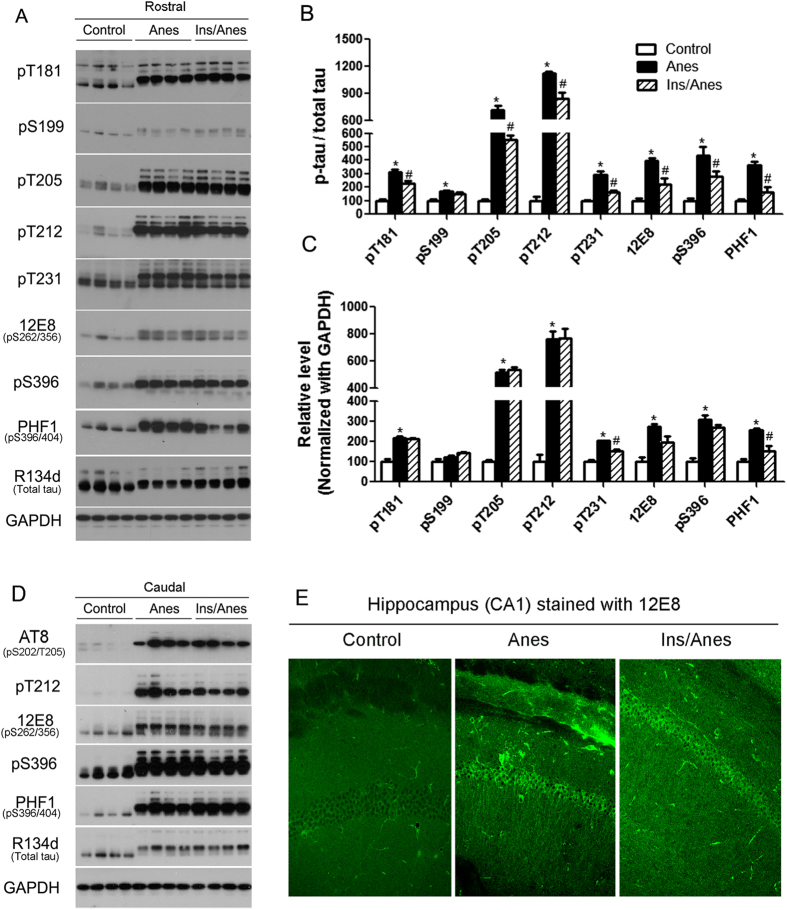
Effect of intranasal insulin and anesthesia on tau phosphorylation. (**A**) Homogenates of the rostral halves of brains of mice sacrificed at the end of anesthesia for one hour were analyzed by Western blots developed with antibody R134d against total tau and several phosphorylation-dependent and site-specific tau antibodies. (**B,C**) Densitometrical quantifications (mean ± SEM) of the blots after normalized with the corresponding total tau levels (**B**) or with the GAPDH levels (**C**). The levels of control group were set as 100. *p < 0.05 vs. control. ^#^p < 0.05 vs. Anes group. (**D**) Homogenates of the caudal halves of forebrains of mice sacrificed at the end of anesthesia for one hour were analyzed by Western blots developed with antibodies indicated at the left of the blots. (**E**) Immunohistochemical staining of the sagittal sections of the mouse brains with monoclonal antibody 12E8. The hippocampal CA1 sector is shown. These results indicate that intranasal insulin attenuates anesthesia-induced hyperphosphorylation of tau in the mouse brain.

To understand the biological changes underlying the cognitive impairment induced by anesthesia and the preventive effect of insulin, we sacrificed the mice after Morris water maze test and analyzed tau phosphorylation in the brain because tau hyperphosphorylation is crucial to AD and other tauopathies^26^. In contrast to previous reports by us^8^,^24^ and others^27^,^28^, we found no detectable change in tau phosphorylation in the brains of anesthetic mice with or without prior treatment with insulin (data not shown). Because the animals were sacrificed within a few hours after anesthesia in all previous studies in which tau hyperphosphorylation was observed post anesthesia, we thus treated three additional groups of mice with intranasal insulin and/or anesthesia, as described above, and sacrificed the mice immediately after anesthesia for one hour and before they awoke from anesthesia. Analysis of brain tau phosphorylation of these mice showed that anesthesia with propofol/sevoflurane induced marked increase in tau phosphorylation at all phosphorylation sites studied (Fig. 2), which are consistent with previous studies using different paradigms of anesthesia^27^,^28^. Up-shift of the apparent gel mobility of tau proteins, which is a well-established phenomenon of tau hyperphosphorylation, was also seen in the Western blots (Fig. 2A). Excitingly, we found that prior treatment of the mice with intranasal insulin attenuated anesthesia-induced tau hyperphosphorylation at most of the sites studied, including Thr181, Thr205, Thr212, Thr231, Ser262/Ser356 (12E8 sites), Ser396, and Ser396/Ser404 (PHF-1 sites) (Fig. 2B,C). The rostral halves of the forebrains were first analyzed because higher insulin concentration can be reached to this area than the caudal part of the brain through intranasal administration^29^.

Because the hippocampus, which is the major brain area responsible for spatial learning and memory, is localized in the caudal half of the mouse forebrain, we also studied tau phosphorylation in the homogenates of the caudal halves of the mouse brains. We found marked increase in tau phosphorylation at all phosphorylation sites studied and an up gel-mobility shift in the caudal halves of the mouse forebrains immediately after anesthesia (Fig. 2D), which is similar to the changes observed in the rostral halves of the mouse brains. Partial prevention of the anesthesia-induced increase in tau phosphorylation was also observed after the pre-treatment of mice with intranasal insulin, but the attenuating effect in the caudal forebrains was less remarkable than in the rostral forebrains. These regional differences are consistent to the higher drug concentration in the rostral forebrain than the caudal forebrain after intranasal administration^29^. The effects of anesthesia and intranasal insulin were clearly detectable in the hippocampal neurons through immunohistochemical staining with monoclonal antibodies 12E8 (Fig. 2E) and PHF1 (data not shown), both of which are against phosphorylated tau.

### Intranasal insulin enhances the level of synaptic proteins in the mouse brain

**Figure 3:**
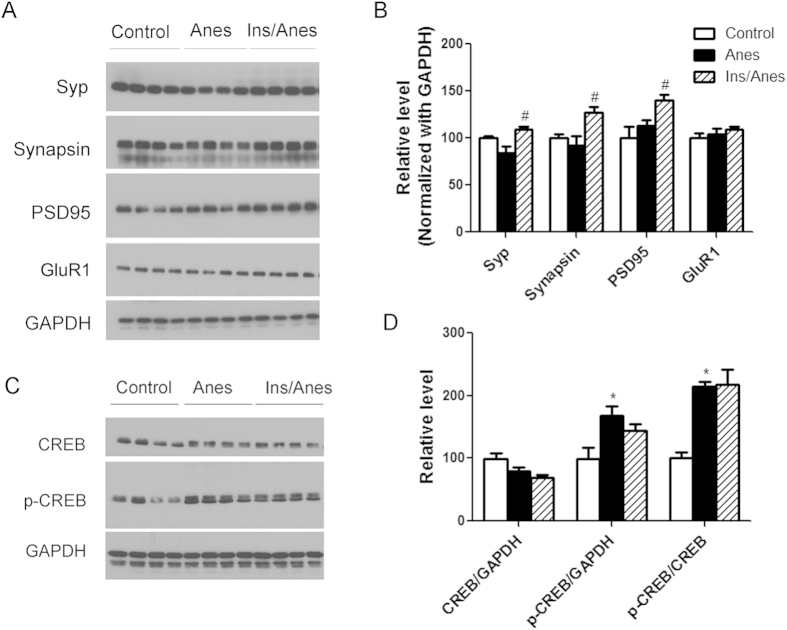
Effect of intranasal insulin and anesthesia on synaptic proteins. (**A,C**) Homogenates of the rostral halves of brains of mice sacrificed at the end of anesthesia for one hour were analyzed by Western blots developed with antibodies indicated at the left side of the blots. (**B,D**) Densitometrical quantifications (mean ± SEM) of the blots. *p < 0.05 vs. control. ^#^p < 0.05 vs. Anes group. These results indicate that intranasal insulin enhances the level of synaptic proteins in the mouse brain.

Synapses are the structural basis of memory and cognition, and their alterations usually underlie functional changes of the brain. To learn whether anesthesia and intranasal insulin treatment alter synaptic activity, we determined the levels of major synaptic proteins in the mouse brains after the treatments using Western blots. These synaptic marker proteins include the presynaptic proteins synaptophysin (Syp) and synapsin-1, the postsynaptic marker postsynaptic density 95 (PSD95), the AMPA receptor subunit GluR1. We found that anesthesia with propofol and sevoflurane did not alter the levels of these synaptic proteins significantly, but pretreatment of mice with intranasal insulin significantly increased the levels of synaptophysin, synapsin-1 and PSD95 by approximately 10–40% in the brains of the anesthesia-treated mice (Fig. 3A,B).

CREB is a transcription factor crucial to neuronal plasticity and long-term memory formation in the brain. Its transcriptional activity is mainly regulated by its phosphorylation^30^. We found that both the level of phosphorylated CREB, as evidenced by the p-CREB/GAPDH, and the net phosphorylation of CREB, as evidenced by the p-CREB/CREB, were increased by approximately 70% and 115%, respectively, after anesthesia, but pre-treatment with intranasal insulin did not induce further changes (Fig. 3C,D). These results suggest that anesthesia with propofol and sevoflurane activate CREB activity.

### Intranasal insulin promotes brain insulin signaling

**Figure 4:**
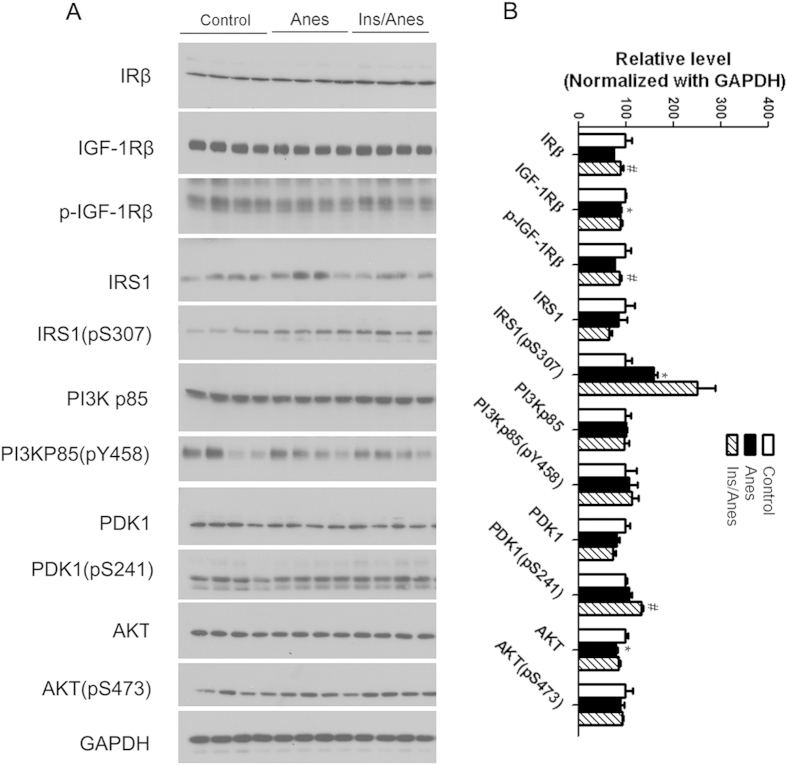
Effect of intranasal insulin and anesthesia on the brain insulin signaling pathway. (**A**) Homogenates of the rostral halves of brains of mice sacrificed at the end of anesthesia for one hour were analyzed by Western blots developed with antibodies indicated at the left side of the blots. (**B**) Densitometrical quantifications (mean ± SEM) of the blots. *p < 0.05 vs. control. ^#^p < 0.05 vs. Anes group. These results suggest that intranasal insulin promotes brain insulin signaling.

We investigated the effect of intranasal insulin treatment and anesthesia on brain insulin signaling by determining the level and activation of each component of the signaling pathway, including insulin receptor β (IRβ), insulin-like growth factor-1 receptor β (IGF-1Rβ), insulin receptor substrate-1 (IRS-1), phosphatidylinositide 3-kinases (PI3K), 3-phosphoinositide-dependent protein kinase-1 (PDK1) and protein kinase B (AKT). The activation of these proteins was assessed by measuring their phosphorylation levels at the activity-dependent sites. We found that brain insulin signaling was somewhat disturbed in mice post anesthesia, as evidenced by significant reduction the levels of IGF-1Rβ and AKT in the anesthesia group as compared to the control group (Fig. 4). We also found that the prior treatment of mice with intranasal insulin increased the levels of IRβ, p-IGF-1Rβ, and p-PDK1 (pS241) in the brains of the anesthesia-treated mice (Fig. 4). These results suggest that intranasal insulin treatment can promote brain insulin signaling.

### Anesthesia and insulin induce transient brain biochemical changes in the brain

**Figure 5:**
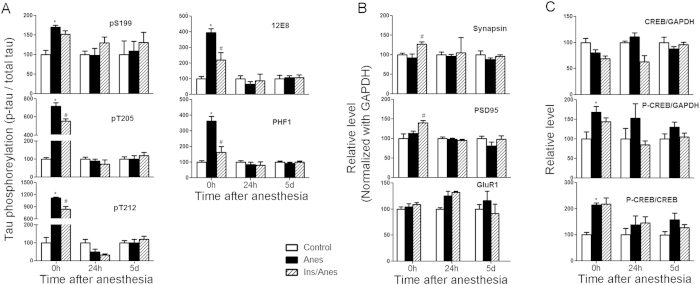
Biochemical changes in the brains of mice post intranasal insulin and anesthesia. Homogenates of the rostral halves of brains from mice sacrificed 0 hr, 24 hr, and 5 days after anesthesia for one hour with or without prior treatment with intranasal insulin were analyzed by Western blots, followed by densitometrical quantifications (mean ± SEM). The levels of the control group were set as 100. *p < 0.05 vs. control. ^#^p < 0.05 vs. Anes group. These results suggest that anesthesia- and insulin-induced biochemical changes in the brain are transient and reversible.

Because the anesthesia-induced hyperphosphorylation of tau was seen immediately after anesthesia but not 5 days later after completion of the probe test in the Morris water maze, we studied how long the anesthesia-induced brain biochemical changes and the intranasal insulin’s effects last by investigating the brains of mice sacrificed 0 hr, 24 hrs, and 5 days after anesthesia. We found increased tau phosphorylation and changes of CREB phosphorylation, as well as insulin’s protective role, only when the mice were sacrificed immediately after anesthesia (Fig. 5). None of the anesthesia-induced changes nor insulin’s effects were seen in the brains of mice sacrificed 24 hrs or 5 days after anesthesia. To learn whether increased tau phosphorylation is still detectable in selected neurons of mice 5 days after anesthesia, we stained the brain sections immunohistochemically with antibodies 12E8 and PHF-1 against phosphorylated tau. However, we did not find any neurons showing significant increase in the immunostaining (data not shown). These results indicate that although the behavioral impacts of anesthesia and insulin were detectable five days post anesthesia, the biochemical brain changes disappear within 24 hrs after anesthesia.

## Discussion

It is well known that general anesthesia can impair short-term memory and also increase the risk for long-term cognitive decline and dementia, especially for vulnerable individuals such as the elderly^1^,^5^. Anesthesia’s adverse impact on cognition has also been replicated in animals^9^,^12^,^13^,^24^. To our knowledge, there is no treatment available to date to prevent the cognitive impairment that occurs in individuals post anesthesia. In the present study using mice as an experimental model, we found that intranasal administration of insulin can prevent cognitive impairment, as well as biochemical changes in the brain, induced by general anesthesia. These findings indicate a potential to use intranasal insulin for preventing anesthesia-induced cognitive impairment in vulnerable individuals after surgery.

Besides epidemiological studies, previous studies have demonstrated that the use of various anesthetics induces hyperphosphorylation of tau in mice^8^,^24^,^27^,^28^. Tau hyperphosphorylation is known to promote neurodegeneration in AD^26^. A few studies also showed cognitive impairment of rodents after anesthesia^9^,^12^,^13^,^28^. In order to make the present study more clinically relevant, we used propofol to induce and sevoflurane to maintain anesthesia because the combinational use of these two anesthetics are commonly used in clinical practice for surgery. Aged mice (17–18-months old) were used in this study because elderly individuals are more vulnerable to anesthesia-induced cognitive impairment. With this anesthetic paradigm, we found that anesthesia induced impairment of spatial learning and memory, which is consistent with previous studies using different anesthetic paradigms^9^,^12^,^13^,^28^.

In a recent study, we found that treatment of 3xTg-AD mice with daily intranasal administration of insulin for 7 days prior to anesthesia with propofol promotes brain insulin signaling and attenuates propofol-induced hyperphosphorylation of tau^24^. We therefore tested whether the same pretreatment with intranasal insulin could also prevent these brain changes induced by a combined use of propofol and inhaled anesthetic in aged non-transgenic mice. More importantly, we were eager to learn whether the insulin pretreatment can prevent anesthesia-induced cognitive impairment. We found in the present study that the pretreatment effectively prevented anesthesia-induced cognitive impairment. It also enhanced the levels of synaptic proteins and brain insulin signaling, as well as partially prevented anesthesia-induced increase of tau phosphorylation in the aged wild-type mice. It is possible administration of daily intranasal insulin for less than seven days may be sufficient to prevent anesthesia-induced cognitive impairment.

Previous studies have reported increased tau phosphorylation and other biochemical changes in the brain immediately after general anesthesia^8^,^24^,^27^,^28^, but how long these anesthesia-induced brain changes last after animals wake from anesthesia remains elusive. In the present study, we found that these brain changes were transient and returned to the normal levels within 24 hrs post anesthesia. These observations are consistent with an early report by Ikeda *et al.*^31^ showing that anesthesia-induced increase in tau phosphorylation peaks at 0–10 min after ether exposure and is reversed to the normal level in 40–60 min. However, the cognitive impairment induced by anesthesia appears to last much longer, because we clearly found impairment in spatial memory in Morris water maze 5 days after anesthesia, when the brain biochemical changes had disappeared. Apparently, the anesthesia-induced transient molecular changes have a much longer functional impact to cognition. Alternatively, other molecular changes induced by anesthesia but not studied in the present study might last long and underlie the cognitive impairment.

To search for a method to prevent anesthesia-induced brain changes and cognitive impairment, we selected insulin in our recent^24^ and the present study because it is a neurotrophic factor and is important to neuroplasticity and cognition^18^. Furthermore, brain insulin signaling is deregulated in the brains of individuals with AD^32^,^33^, in which progressive cognitive impairment and dementia are the major clinical symptoms. Systemic administration of insulin is obviously undesirable because it is difficult to enter into the brain and would otherwise disturb metabolism in the periphery and may lead to hypoglycemia. Intranasal administration, which was first introduced by W. H. Frey, bypasses the blood brain barrier and has been used successfully in animal studies and clinical trials in humans^21^,^34^,^35^,^36^,^37^,^38^. Intranasal administration of insulin does not appear to interfere with the insulin level or glucose metabolism in the periphery^21^. This insulin delivery method is simple and non-invasive and readily applicable in clinic. Our findings reported in the present study suggest that simply administering insulin into the nostrils of patients before anesthesia may prevent anesthesia’s adverse effect on cognition. Future studies will determine the minimal dose and the best time before anesthesia for intranasal insulin administration.

The underlying molecular mechanism by which intranasal insulin prevents anesthesia-induced cognitive impairment remains to be elucidated. Anesthesia might promote cognitive impairment and AD by promoting Aβ production^10^,^11^,^39^, tau hyperphosphorylation^8^,^27^,^28^,^40^, and neuroinflammation^41^. The present study demonstrates that intranasal insulin can prevent anesthesia-induced tau hyperphosphorylation and promote the expression of synaptic proteins. Although brain Aβ level was not determined in the present study, we found in a recent study that intranasal insulin reduces Aβ level in the brains of 3xTg-AD mice and inhibits microglial activation^40^. Therefore, intranasal insulin might prevent anesthesia’s adverse effect on cognition through inhibiting tau hyperphosphorylation, neuroinflammation and synaptic damage induced by anesthesia. One mechanism by which anesthesia induces tau hyperphosphorylation is through down-regulation of protein phosphatase 2A (PP2A)^7^,^42^. By using 3xTg-AD mice, we recently found that intranasal insulin up-regulates PP2A in the anesthetized mouse brains, as evidenced by increased levels of both the total and the methylated form (more active form) of the catalytic subunit of PP2A^24^. In the present study using aged wild-type mice, we also studied the catalytic subunit of PP2A and its methylation by Western blots in the mouse brains and found that intranasal insulin treatment increased PP2A level and its methylation (data not shown). Therefore, intranasal insulin administration could prevent anesthesia-induced tau hyperphosphorylation partially through upregulation of PP2A.

In conclusion, we report here that intranasal administration of insulin prior to anesthesia can prevent spatial learning and memory impairment and increased tau phosphorylation induced by general anesthesia induced by propofol and sevoflurane. These findings provide a simple strategy, i.e., administering insulin into the nose before anesthesia, to prevent postoperative cognitive deficit and increased risk for developing AD and dementia induced by general anesthesia.

## Materials and Methods

### Antibodies and reagents

Primary antibodies used in this study are listed in Table 1. Peroxidase-conjugated anti-mouse and anti-rabbit IgG were obtained from Jackson ImmunoResearch Laboratories (West Grove, PA, USA). The enhanced chemiluminescence (ECL) kit was from Pierce (Rockford, IL, USA). The ABC staining system was from Santa Cruz Biotechnology (Santa Cruz, CA, USA). Propofol was purchased from MP Biomedicals (Solon, OH, USA). Insulin (Humulin R U-100) was from Eli Lily (Indianapolis, IN, USA). Other chemicals were from Sigma-Aldrich (St. Louis, MO, USA).

### Animals and animal treatments

The breeding pairs of the C57BL6/129 mice were initially obtained from Jackson Laboratory (New Harbor, 124 ME, USA), and the mice were bred in our institutional animal colony. Mice were housed (4 ∼ 5 animals per cage) with a 12/12 h light/dark cycle and with ad libitum access to food and water. The housing, breeding, and animal experiments were in accordance with the approved protocol from the Institutional Animal Care and Use Committee of New York State Institute for Basic Research in Developmental Disabilities, according to the PHS Policy on Human Care and Use of Laboratory animals (revised March 15, 2010). Female mice at the ages of 17–18 months were used for this study. Aged mice were chosen because elderly individuals are more vulnerable to anesthesia-induced cognitive impairment.

Intranasal delivery was carried out manually without anesthesia while the mouse head was restrained in a supine position with the neck in extension, as described^36^. A total of 1.75 U/17.5 μl insulin or 0.9% saline was delivered over both nares alternatively using a 10 μl Eppendorf pipetter. The mouse was held for an additional 5–10 seconds to ensure the fluid was inhaled. The successful nasal delivery by using this approach was confirmed by examination of ink (Fount India ink, Pelikan, Schindellegi, Switzerland) in the autopsied brains after nasal delivery with ink using the same approach (data not shown). All mice were treated with insulin or, as a control, saline daily for 7 consecutive days (Fig. 1A). On the following day, the mice were injected intraperitoneally (i.p.) with propofol dissolved in intralipid (150 mg/kg body weight) or the equivalent amount of intralipid, followed by inhalation of 2.5% sevoflurane for 1 hr. Mice were sacrificed immediately, 24 hrs or 5 days post anesthesia by cervical dislocation. The mouse forebrains were removed immediately and divided into two hemispheres. The left hemispheres were fixed in 4% paraformaldehyde in 0.1 M phosphate buffer for immunohistochemical studies. The right hemispheres were further divided into rostral and caudal halves (separated coronally at the bregma level), flash frozen in dry ice, and stored at −80 °C for biochemical analyses at a later date.

### Morris Water Maze

Morris water maze (MWM) was used to evaluate spatial learning and memory of the mice. The test was performed in a circular white pool (with a diameter of 180 cm and a height of 60 cm) filled with white dye tinted water and maintained at room temperature (20 ± 1 °C). The maze was designated of two principal axes with each line bisecting the maze perpendicular to the other one to divide the maze into four equal quadrants. The end of each line demarcates four cardinal points: north (N), south (S), east (E) and west (W). A platform was positioned in the middle of one of the quadrants submerged 1 cm below water surface. Each mouse performed 4 trials per day for 4 consecutive days from semi-random start positions to find the hidden platform. Each trial was terminated as soon as the mouse climbed onto the hidden platform. If a mouse failed to find the platform within 90 sec, it was gently guided to it. At the end of each trial, the mouse was left on the platform for 20 sec, then dried and returned to its home cage. A 60 sec probe test without platform was performed 24 hr after the last trial. The swim path, swim distance (cm), escape latency (sec), swim speed (cm/sec), time spent in each quadrant (sec), distance traveled in each quadrant (cm), latency to enter the platform site zone (sec), and the number of platform site zone crossings were recorded through an automated tracking system (Smart video tracking system, Panlab; Havard Apparatus).

### Western blot analysis

Brain tissue was homogenized in pre-chilled buffer containing 50 mM Tris-HCl (pH7.4), 50 mM GlcNAc, 20 μM UDP, 1.0 mM EGTA, 2 mM Na_3_VO_4_, 100 mM NaF, 0.5 mM AEBSF, 1 μg/ml aprotinin, 10 μg/ml leupeptin, and 2 μg/ml pepstatin A. Protein concentrations of the homogenates were determined by the Pierce 660-nm Protein Assay. The samples were resolved in 10% or 12.5% SDS-PAGE and electrotransferred onto Immobilon-P membrane (Millipore, Bedford, MA, USA). The blots were then probed with primary antibody and developed with the corresponding horseradish peroxidase-conjugated secondary antibody and ECL kit.

### Immunohistochemistry

Frozen mouse brain sagittal sections (40-μm thick) were first washed with phosphate-buffered saline (PBS) for three times, 15 min each, followed by incubation in 0.5% Triton X-100 for 20 min. The sections were then washed with PBS for another 10 min and then blocked in PBS containing 5% normal goat serum and 0.1% Triton X-100 for 30 min, followed by incubation with 12E8 or PHF1 in the blocking solution at 4 °C overnight. After washing with PBS, the sections were incubated with Alexa 488-conjugated goat anti-mouse IgG (1:1000) plus TO-PRO-3 in the blocking solution at room temperature for 2 hrs. The sections were finally washed, mounted, and cover slipped using Prolong^®^ gold antifade mountant (Invitrogen, Carlsbad, USA). The immunostaining was analyzed by using a laser scanning confocal microscope (PCM 200, Nikon).

### Statistical analysis

For biochemical analyses, data were analyzed by one-way ANOVA followed by Tukey’s post hoc tests or unpaired two-tailed *t* tests, using GraphPad. All data are presented as means ± SEM, and *p* < 0.05 was considered statistically significant.

## Additional Information

**How to cite this article**: Zhang, Y. *et al.* Intranasal Insulin Prevents Anesthesia-Induced Spatial Learning and Memory Deficit in Mice. *Sci. Rep.* **6**, 21186; doi: 10.1038/srep21186 (2016).

## Figures and Tables

**Table 1:** Primary antibodies used in this study.

Antibody	Type	Specificity	Phosphorylation sites	Source/Reference
IRβ	Poly-	IRβ		Cell Signaling Technology, Danvers, MA
IGF-1Rβ	Poly-	IGF-1Rβ		Cell Signaling Technology
P-IRβ/IGF-1Rβ	Mono-	P-IRβ/IGF-1Rβ	Tyr1150/1151(IRβ), Tyr1135/1136 (IGF-1Rβ)	Cell Signaling Technology
IRS1	Poly-	IRS1		Cell Signaling Technology
IRS1 pS307	Poly-	P-IRS1	Ser307	Cell Signaling Technology
PI3K p85	Poly-	PI3K (p85)		Cell Signaling Technology
P-PI3K p85	Poly-	P-PI3K (p85)	Tyr458/Tyr199	Cell Signaling Technology
PDK1	Poly-	PDK1		Cell Signaling Technology
PDK1 pS241	Poly-	P-PDK1	Ser241	Cell Signaling Technology
AKT	Poly-	AKT		Cell Signaling Technology
AKT pS473	Poly-	P-AKT	Ser473	Cell Signaling Technology
AKT pT308	Poly-	P-AKT	Thr308	Cell Signaling Technology
R134d	Poly-	Tau		Tatebayashi *et al.*, 2012^43^
pT181	Poly-	P-tau	Thr181	Invitrogen, Grand Island, NY
pS199	Poly-	P-tau	Ser199	Invitrogen
pT205	Poly-	P-tau	Thr205	Invitrogen
pT212	Poly-	P-tau	Thr212	Invitrogen
pT231	Poly-	P-tau	Thr231	Invitrogen
pS396	Poly-	P-tau	Ser396	Invitrogen
12E8	Mono-	P-tau	Ser262/356	Dr. D. Schenk^44^
AT8	Mono-	P-tau	Ser202/T205	Thermo Fisher Scientific, Rockford, IL
PHF-1	Mono-	P-tau	Ser396/404	Dr. P. Davies^45^
pS409	Poly-	P-tau	Ser409	Invitrogen
Synapsin-1	Poly-	Synapsin-1		Santa Cruz Biotechnology
Synaptophysin	Mono-	Synaptophysin		Millipore
PSD95	Mono-	PSD95		Cell Signaling Technology
GluR1	Poly-	GluR1		Millipore
GFAP	Mono-	GFAP		Millipore
ED1	Mono-	CD68		Abcam
CREB	Mono-	CREB		Cell Signaling Technology
p-CREB	Mono-	p-CREB	Ser133	Cell Signaling Technology
Anti-GAPDH	Poly-	GAPDH		Santa Cruz Biotechnology
